# Recurrent Spontaneous Coronary Artery Dissection as the Cause of Repeated Myocardial Infarctions

**DOI:** 10.1002/ccr3.70083

**Published:** 2025-01-10

**Authors:** Arnon Møldrup Knudsen, Nicolaj Brejnholt Støttrup, Henrik Hager, Henning Mølgaard, Christina Stilling

**Affiliations:** ^1^ Department of Pathology Aarhus University Hospital Aarhus Denmark; ^2^ Department of Cardiology Aarhus University Hospital Aarhus Denmark

**Keywords:** infarction sequels, myocardial infarction, myocardial reperfusion injury, spontaneous coronary artery dissection

## Abstract

Spontaneous coronary artery dissection (SCAD) is characterized by intramural hematoma in a coronary artery leading to partial or complete vessel obstruction. A 51‐year‐old female was hospitalized with acute myocardial infarction and cardiogenic shock. She was diagnosed with severe SCAD, affecting the proximal left coronary artery. A complex percutaneous coronary intervention, complicated by cardiac arrest and need for cardio pulmonary support, succeeded with stent insertion and revascularization. In the following days, the patient developed severe heart failure due to extensive cardiac reperfusion injury and subsequently experienced multiple organ failure, ultimately resulting in death. The patient had previously been acutely hospitalized twice with myocardial infarctions and both the times was also diagnosed with SCAD affecting the left coronary artery. This case highlights an unfortunate patient outcome due to recurrent SCAD and serves as an important reminder to consider SCAD differential diagnostically in younger female patients with myocardial infarction.

## Graphical abstract

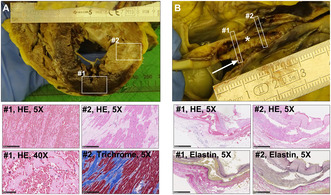

**Summary**

- Spontaneous coronary artery dissection is a relatively rare cause of myocardial infarction among the general population, but is an important differential diagnosis in younger female patients without cardiovascular risk factors and in pregnant individuals presenting with acute coronary syndrome.

## 1 Introduction

Spontaneous coronary artery dissection (SCAD) is an intramural loosening and bleeding of the tunica media within a coronary artery wall that can lead to reduced blood flow or complete luminal arterial obstruction. The clinical presentation varies from short‐term chest discomfort to severe chest pain and acute coronary syndrome, with risk of myocardial infarction [1]. The prevalence of SCAD among all patients hospitalized with acute coronary syndrome is approximately 4% [2], while up to 90% of all SCAD cases are seen among younger females aged 43–57, with no prior cardiovascular risk factors [3]. SCAD most commonly affects the left anterior descending (LAD) coronary artery [4]. The etiology is multifactorial, and known predisposing factors include underlying vessel disease or connective tissue disease such as fibromuscular dysplasia, various genetic syndromes such as Marfan–Ehlers–Danlos, Loeys–Dietz, and Alport syndromes, as well as hormonal fluctuations including pregnancy, hypertension, and systemic inflammatory conditions. In predisposed individuals, SCAD can be triggered and manifest after physical or emotional stress [5].

### 1.1 Case History/Examination

**FIGURE 1:**
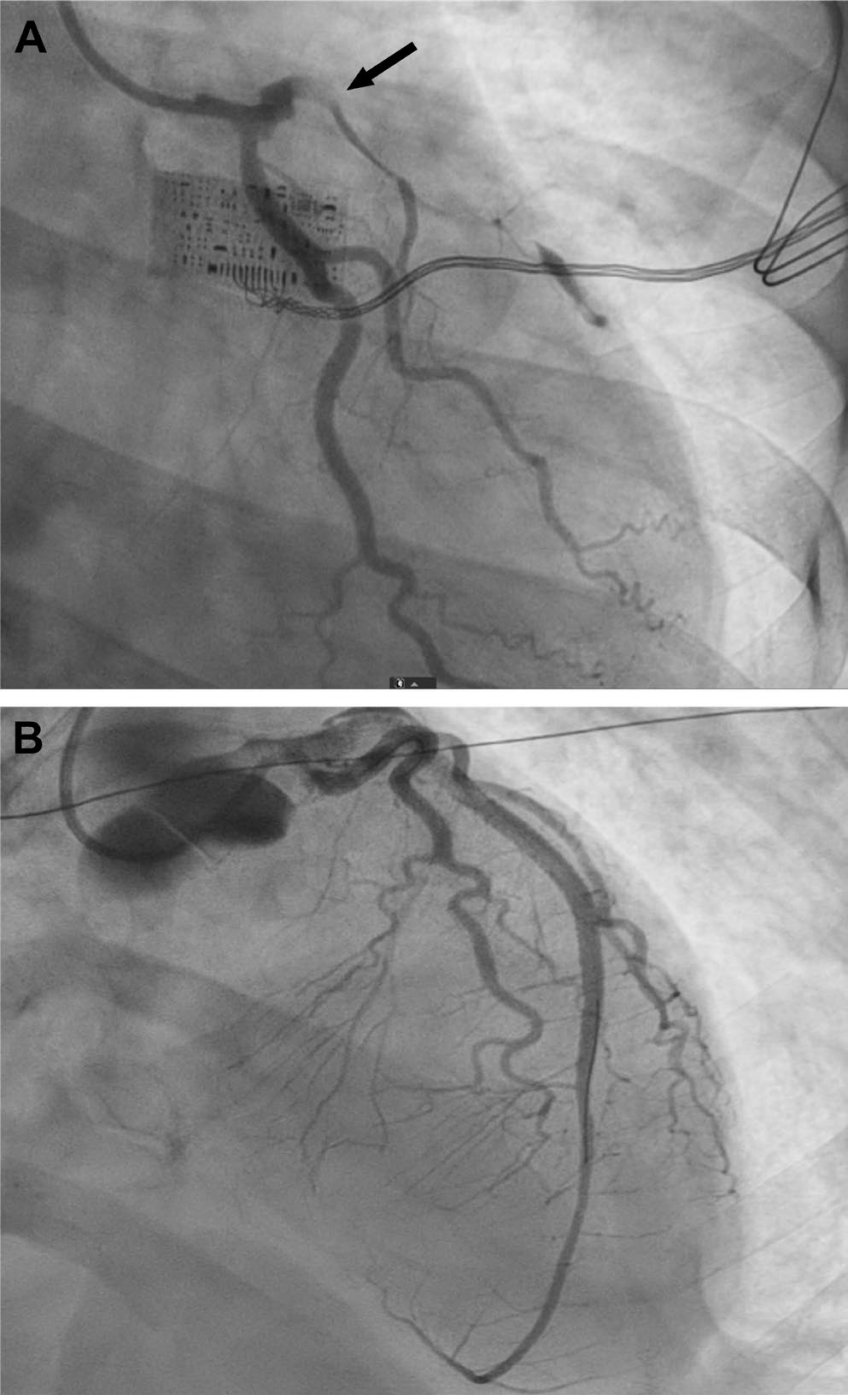
Coronary angiography images before and after percutaneous intervention. (A) Initially, SCAD was identified in the left main coronary artery and the LAD branch, as evident by luminal narrowing and impaired passage of intravascular contrast (Black arrow). (B) After the complicated percutaneous coronary intervention with successful insertion of stents was completed, TIMI‐3 luminal blood flow was restored, and the LAD branch showed homogenous contrast distribution throughout its full extent.

A 51‐year‐old female experienced acute severe chest pain and developed bystander witnessed out‐of‐hospital cardiac arrest. Basic life support was immediately initiated, and upon arrival of paramedic staff, an ECG showed ventricular fibrillation. Resuscitation was successful, and return of spontaneous circulation was achieved shortly prior to arrival at the cardiac laboratory, where the patient was initially stabilized. An echocardiography showed an ejection fraction of approximately 25% and regional hypokinesia in the supply area of LAD. The following coronary angiography revealed pronounced SCAD with dissection of the left main coronary artery and LAD branch (Figure 1A). The patient was discussed ad hoc with thoracic surgeons, but deemed unfit for surgery.

## 2 Methods (Differential Diagnosis, Investigations, and Treatment)

A complicated percutaneous coronary intervention was performed, during which the patient experienced renewed ventricular fibrillation after formation of a spontaneous embolus in the circumflex artery. The patient was connected to an extra corporal membrane oxygenation system for circulatory support, and stents were successfully inserted into the left coronary artery and LAD, resulting in restoration of TIMI‐3 blood flow (Figure 1B). Later, the ejection fraction was estimated to approximately 10% and Troponin I measured to > 1.5 million ng/L.

In the following days, the patient had unchanged severe heart failure, with an ejection fraction remaining at 10%, leading to insufficient organ perfusion and accumulation of lactic acid. The patient eventually developed multi‐organ failure despite maximal treatment efforts, and ultimately the treatment was ceased, with the patient passing away shortly thereafter.

## 3 Conclusion and Results

**FIGURE 2:**
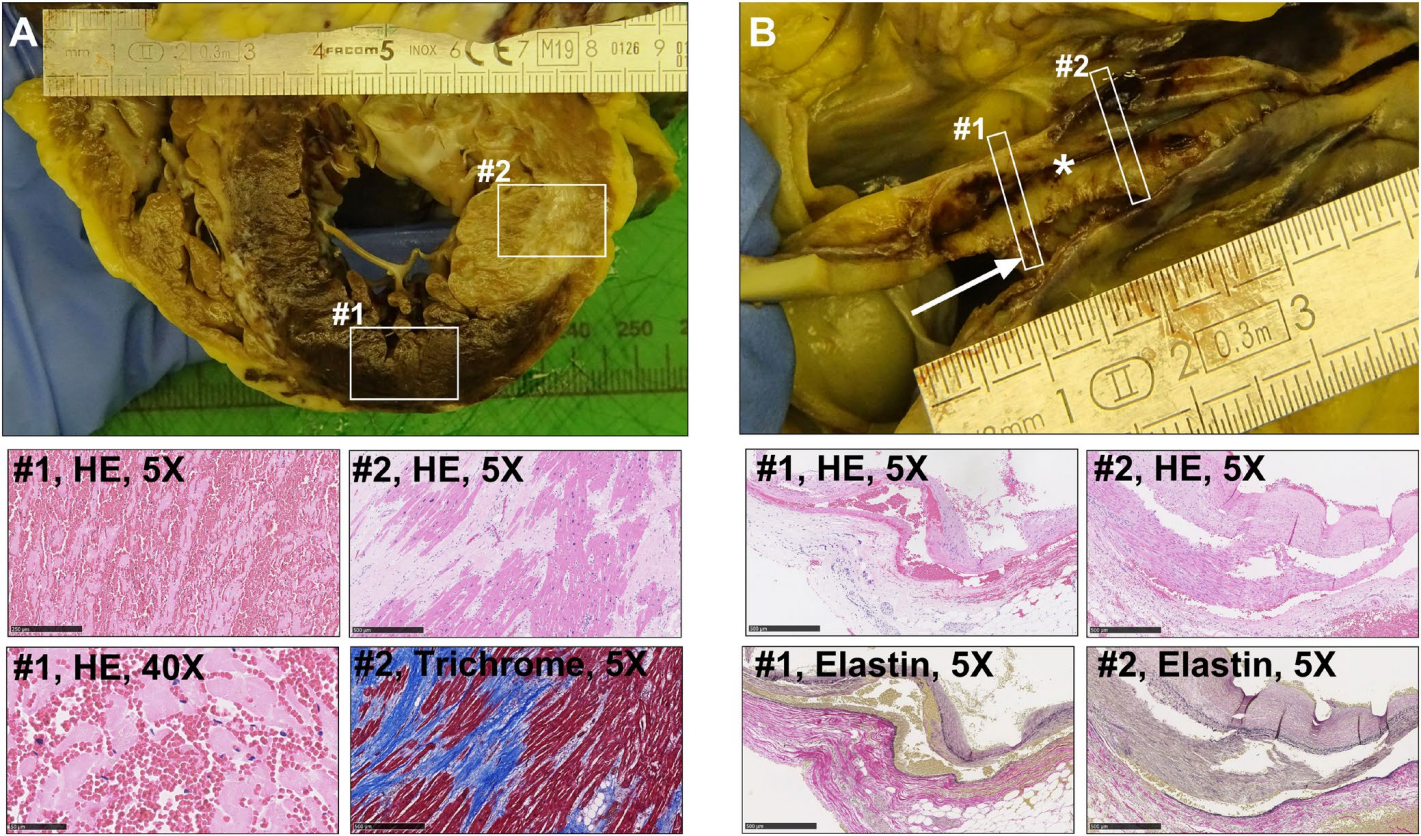
Postmortem pathological findings. (A) Photo depicting a full cross section of the heart revealing pronounced transmural sequels of myocardial infarction in the left ventricle and septum. Interstitial bleeding and cardio myocyte necrosis with loss of nuclei is represented in the microscopy image from region #1. The infarction involves both the septal and diagonal branches of LAD supplying the anterior two‐thirds of septum and the anterior–lateral part of the left ventricle, respectively. Further, fibrosis as a sequel from a previous myocardial infarction is evident in the HE section and also seen as strong blue staining in the supplementary Mason's trichrome stain (representative image from region #2). (B) Photograph depicting the left coronary artery and LAD, where all sampled sections (representative images from region #1 + 2) show pronounced dissection of the arterial wall with intramural bleeding of the tunica media, as illustrated on HE sections and Verhoeff elastin stains. The white arrow indicates the true vessel lumen, while the white star shows the false lumen created by the dissection. Scale bars = 500 μm at 5× magnification and 50 μm at 40× magnification.

An autopsy was performed, and in the heart the left ventricular wall was hypertrophic. The myocardium in the antero‐lateral part of the left ventricle and the anterior two‐thirds of septum was characterized by severe transmural reperfusion injury (Figure 2A). In the same region, fibrotic tissue was identified, indicating previous infarction. In the left main coronary artery and LAD, the SCAD changes were located and confirmed (Figure 2B). In all coronary arteries, only mild atherosclerotic changes in the form of fatty streaks were identified.

The patient had previously, at age 39, been admitted to emergency care with a non‐ST‐elevation myocardial infarction, where a coronary angiography showed SCAD in a branch of the LAD resulting in 50% stenosis. At age 48, she was again acutely hospitalized with severe chest pain and was diagnosed with an ST‐elevation myocardial infarction. Coronary angiography showed SCAD from the middle part of the LAD involving the distal half of the artery. The patient was treated conservatively on both occasions, due to spontaneous symptom remission. Following the event, the patient was genetically tested for potential pathogenic gene variants related to aortic vascular disease. This was performed due to possible familial disposition, given a twin sister also diagnosed with SCAD, and her father and paternal aunt both having aortic aneurysms. However, no pathogenic genetic variants were identified.

Postmortem and, supplementary, more extensive genetic testing of 106 genes involved in hereditary cardiomyopathies and familial hypercholesterolemia, were performed. The testing included, among others, the genes *ACTC1, MYBPC3, MYH7, MYL2, MYL3, TNNI3*, and *TNNT2* involved in hypertrophic cardiomyopathy [6]; *DSP, LDB3, LMNA, PLN, RBM20, SCN5A*, and *TTN* involved in dilated cardiomyopathy [7]; and *APOB, LDLR*, and *PCSK9* involved in familial hypercholesterolemia [8]. No pathogenic or likely pathogenic variants were identified.

## 4 Discussion

Although SCAD is a relatively rare cause of myocardial infarction among the general population, it is estimated to be the cause of up to 35% of infarctions in females younger than 50 years [2]. Further, SCAD is the most common cause of myocardial infarction among pregnant women and in the postpartum period and should be excluded in case of acute chest pain in this specific patient group [9].

SCAD recurs in approximately 10% of patients, and the recurrence risk increases with untreated or insufficiently treated hypertension [10]. Given the relatively low recurrence risk, a presentation as reported in this case, with multiple recurrences leading to several myocardial infarctions, and ultimately death, is unusual and a reminder of the potential severity of SCAD, which is usually treated conservatively [11].

The reported prevalence of SCAD patients with simultaneous vascular and/or connective tissue disease varies significantly, ranging from 30.1% to 80.7% [2]. When SCAD is diagnosed, genetic testing for potential underlying predisposing diseases should therefore be considered. In patients with underlying vascular and/or connective tissue disease experiencing symptoms of myocardial infarction, SCAD should be considered as a differential diagnosis. In this case, supplementary comprehensive genetic testing was performed without positive findings, thereby excluding the most well‐annotated genetic causes of aortic disease, cardiomyopathy, and familial hypercholesterolemia.

Taken together, SCAD is a relatively rare cause of myocardial infarction among the general population; however, in younger and predisposed females < 50 years old, SCAD is an important differential diagnosis as the cause of myocardial infarction.

## Author Contributions

**Arnon Møldrup Knudsen:** conceptualization, data curation, formal analysis, funding acquisition, investigation, methodology, project administration, visualization, writing – original draft. **Nicolaj Brejnholt Støttrup:** data curation, formal analysis, investigation, methodology, resources, validation. **Henrik Hager:** conceptualization, formal analysis, investigation, methodology, supervision. **Henning Mølgaard:** formal analysis, investigation, methodology, supervision, validation. **Christina Stilling:** conceptualization, data curation, formal analysis, investigation, supervision, validation.

## Ethics Statement

Written consent for publication has been obtained from the patient's next of kin.

## Conflicts of Interest

The authors declare no conflicts of interest.

## Data Availability

Data are available from the corresponding author upon reasonable request.
